# Influence of Educational Specialty on Perceptions towards Corporal Expression of Prospective Teachers

**DOI:** 10.3390/children10020337

**Published:** 2023-02-09

**Authors:** Jorge Rojo-Ramos, Antonio Castillo-Paredes, Jose Carmelo Adsuar, María Mendoza-Muñoz, Angel Denche-Zamorano, Santiago Gomez-Paniagua

**Affiliations:** 1 Physical Activity for Education, Performance and Health, Faculty of Sport Sciences, University of Extremadura, 10003 Caceres, Spain; 2 Grupo AFySE, Investigación en Actividad Física y Salud Escolar, Escuela de Pedagogía en Educación Física, Facultad de Educación, Universidad de Las Américas, Santiago 8370040, Chile; 3 Promoting a Healthy Society Research Group (PHeSO), Faculty of Sport Sciences, University of Extremadura, 10003 Caceres, Spain; 4 Research Group on Physical and Health Literacy and Health-Related Quality of Life (PHYQOL), Faculty of Sport Sciences, University of Extremadura, 10003 Caceres, Spain; 5 Departamento de Desporto e Saúde, Escola de Saúde e Desenvolvimento Humano, Universidade de Évora, 7004-516 Évora, Portugal; 6 BioẼrgon Research Group, University of Extremadura, 10003 Cáceres, Spain

**Keywords:** corporal expression, future teachers, gender, education specialty, perceptions

## Abstract

Most educators overlook the importance of corporal expression, even though it has been shown to have various advantages for children of all ages. In the teaching–learning process, teacher views and beliefs are crucial because they have a significant impact on students. Therefore, the purpose of this research is to analyze the existing differences in the perceptions of future teachers towards corporal expression according to their gender and educational specialty. A total of 437 aspiring Spanish instructors participated in the sample, selected by the convenience sampling method, and answered the Questionnaire to Assess Perceptions of Corporal Expression in Future Spanish Teachers to gauge their understanding of and preparation for corporal expression via Google Forms. The Mann–Whitney U test was employed to evaluate the possible differences between the diverse items and factors according to gender and educational specialty. The results displayed good perceptions of corporal expression throughout the sample, finding significant differences in most items and all the dimensions when education specialty is considered. Nevertheless, gender variables did not seem to mediate those perceptions. Therefore, university degrees oriented to education must include the same amount of content related to corporal expression to ensure adequate initial training regardless of the stage of education at which the teachers develop their academic activity.

## 1. Introduction

In recent decades, there has been a resurgence of interest in understanding student and teacher attitudes toward various areas within education [1]. Exploring students’ attitudes towards a subject allows public organisations and teachers to know the reality or current situation of their students in relation to certain content and consequently to make decisions to improve the quality of learning, as it has been shown that favourable attitudes regarding a subject enhance the learning environment, and vice versa [2]. Therefore, it is necessary to generate motivation of the students towards certain educational content to improve the learning process as it allows them to improve their attitudes towards them [3]. Kretschmann and Wrobel [4] define attitude as the main determinant of behaviour, which is governed by two main dimensions, affective (emotions and feelings) and cognitive (beliefs and knowledge) [5], and may be more influenced by one dimension than by the other [6]. It is crucial to comprehend how much attitudes influence behaviour and what procedures should be performed to change them, as the scientific literature demonstrates that attitudes have a significant impact on human behaviour [7].

The most-studied context in terms of attitudes is the school, due to its great amount of possibilities to favour good attitudes, mainly because these dispositions are not innate but are acquired through diverse experiences [8]. Thus, the relationship between teachers and students is another factor to take into account in the generation of positive attitudes, since a bad relationship generates in the child a rejection of school and behavioural problems [9], although it is inevitable that children are influenced by their family and home environment, media, and friends [10]. Various personal traits, such as gender, cultural background, and predispositions, influence teachers’ attitudes [11]; their development is influenced by external circumstances such as interactions with children who have different needs, educational backgrounds, and self-efficacy [12].

Young children’s motor, cognitive, and socioemotional development are all tightly correlated with physical education [13]. The subject of physical education is the best setting for teaching cooperation, healthy levels of competition, and social and personal responsibility [14]. Therefore, schools have to establish a favourable learning environment, encourage children to engage in daily physical activity habits that will last a lifetime, and help them adopt a healthy lifestyle [15]. Actually, when we observe students in schools, we discover that attitudes toward physical education and sports classes are often good, and the possibilities of working with content in this area are even greater [16]. Although the benefits of physical education in primary schools in promoting healthy life habits in children are often emphasized, sadly many classroom teachers try to avoid physical education courses because they lack the confidence, teaching expertise, and time necessary to effectively instruct physical education courses [17].

Corporal expression (CE) is understood as the language that becomes an educational subject and is used for the potential development of the expressive capacity of the human being, promoting personal knowledge, interpersonal communication, and the externalization of the internal feelings of the individual, through gestures, postures, and expressive movements [18]. This content has a globalizing value that allows working and developing physical, intellectual, affective, and social aspects, as well as an integrating character, given that it is not reduced exclusively to perceptive-motor aspects, but joins them to the aspects specified above [19]. All of this is accomplished through games and other ludic activities, which enable individuals or groups to develop positive attitudes and even attain an inclusive education [20]. CE is a teaching tool that allows one to communicate non-verbally through the body, establish positive relationships with the members of the group, and develop the creative capacity of the students [21]. However, despite being part of the content of the area of PE in the Spanish education system, the work carried out by many teachers about this content has often been intuitive, as there is no specific teaching model for its development [18].

Typically, it has been found that the attitudes and beliefs of pre-service teachers were relatively static during teacher preparation [22]; in fact, pre-service teachers come into training programs with ideas about education that, once established over time, are challenging to alter [23]. Therefore, initial teacher education is a crucial time to positively influence the beliefs and attitudes of prospective teachers [24]. More specifically, concerning CE, universities do not usually give space to CE within the academy, and even less so in degrees unrelated to artistic expression [25]. In education, teachers, in general, have received little or no training in CE during university and prior to their training, they have had hardly any experience related to CE, due to its late inclusion in the educational curriculum in Spain [26,27]. Already in some studies, a great percentage of future teachers expressed a clear lack of CE content in their higher education [28] and will therefore have difficulties in the future when it comes to successful teaching and implementing this type of content in the classroom [29]. This leads to the fact that CE is one of the contents that is least worked on in the classroom, giving more importance and a better predisposition to sports and collective games [27,30,31]. Therefore, the treatment of CE in teacher training is especially relevant, as this, together with their previous experiences with respect to CE, could have a great influence on the approach or not of the didactic units of this content, in addition to which an erroneous approach could condition the experiences and attitudes of their future students [1].

Extremadura is one of the Autonomous Communities of Spain, which presents peculiar economic, territorial, and population characteristics that have conditioned it to a slight socio-economic backwardness from the rest of the Spanish regions, with the risk of poverty 10 points above the national average (21.7% vs. 32.3%). At the educational level, Extremadura has progressively increased spending on education since 2013 [32]; however, its university in 2018 had the second-lowest budget per student [33]. Regarding university degrees related to education, (1) although the trend in enrolments is beginning to even out in primary education, all the centres where both primary education and infant education are taught at the University of Extremadura have a majority of female students enrolled [34], (2) the student–teacher ratio is slightly higher in primary education than in early childhood education [34], and (3) in the case of primary education since 2017–2018, it has experienced one of the sharpest declines in student enrolment in a degree course at the University of Extremadura in one of the cities where it is taught (from 676 to 593) [35].

Therefore, the objective of this research is to analyze the perceptions towards CE in future teachers in the Community of Extremadura, Spain, also evaluating differences considering gender and the specialty of their studies. In this way, we intend to characterise the current situation of future teachers in terms of CE, so that their initial training can be modified to improve it. Thus, it was hypothesised that perceptions towards CE among future teachers in the Community of Extremadura differ according to gender and the specialty of their studies.

## 2. Materials and Methods

### 2.1. Participants

The sample selection was carried out following a convenience sampling method [36] and was composed of 437 students (Table 1) from the primary education and early childhood education degree programs from the faculties of Teacher Training and Education of the University of Extremadura.

### 2.2. Procedure

To carry out this study, the collaboration of the professors of the corporal expression area of the Faculty of Teacher Training and the Faculty of Education was requested to inform students in the fourth year of the program in early childhood education and primary education of the objective of the study.

Those teachers who agreed to collaborate informed their students of the objective of the study and provided, through the virtual classroom of their subjects, the informed consent and the URL link to the questionnaire on the perception of CE in future teachers using the Google Forms tool. In order to store all the answers in the same database, it was decided to use an electronic questionnaire that would allow the answers to be automatically dumped and make it easier to administer the instrument to the students, which led to a higher response rate. Before starting all telematics surveys, all participants read the informed consent form and accepted after reading the mandatory field “I have read and agree to participate in the study”. In addition, once the questionnaire was completed, a copy of the consent form was automatically sent to the email address provided by the participant (by activating in Google forms: Send respondents a copy of their response).

The data were collected during September and October 2022 and the average time required to complete the questionnaire was 10 min.

### 2.3. Instruments

In terms of characterizing the sample, a questionnaire was prepared with three sociodemographic questions: gender, specialty of study, and age.

The Questionnaire to Assess Perceptions of Corporal Expression in Future Spanish Teachers was adapted from Arias-García et al. [1] and was validated (Cronbach’s alpha: 0.713 to 0.927) by Rojo-Ramos and colleagues [37] in future Spanish teachers (Table A1; Appendix A). The questionnaire was composed of 23 items grouped into three dimensions. Dimension 1, “Evaluation of Corporal Expression”, is composed of 13 items (from 1 to 13) aimed at analyzing the level of contribution that CE has for them. Dimension 2, “Preference”, is composed of three items (from 14 to 16) that analyze the value that future teachers give to CE compared to other content. Dimension 3, “Pleasure”, analyzes the positive situations that students have towards CE. Responses use a Likert scale from 1 (strongly disagree) to 5 (strongly agree).

Rojo-Ramos et al. [37] reported a Cronbach’s alpha value of 0.873 for the first factor, 0.713 for the second, and 0.927 for the third. The values the authors reported for McDonald’s Omega were 0.884, 0.721, and 0.944 for the three factors, respectively.

### 2.4. Statistical Analysis

With the help of the Statistical Package for Social Sciences, all the data were processed and examined (Version 26, IBM SPSS, Chicago, IL, USA). The assumption of normality was not met; hence, nonparametric statistical tests were applied after the Kolmogorov–Smirnov test was used to examine the distribution of the data.

For continuous variables, data are presented as mean and standard deviation and for categorical variables, as numbers and percentages. The Mann–Whitney U test was used to analyze the differences in the scores obtained by the participants according to sex and specialty of study in each of the dimensions of the instrument as well as in each of the items. The Bonferroni correction was applied, and a significance level was established for the *p*-value of <0.001 for the analysis of each of the items and <0.016 for each of the dimensions.

Finally, Cronbach’s alpha was used to analyze the reliability of each of the dimensions. To interpret the level of reliability obtained, Nunnally and Bernstein [38] were used as a reference, indicating that values above 0.70 can be considered satisfactory.

## 3. Results

Table 2 shows the scores obtained in each of the items of the Questionnaire to Assess Perceptions of Corporal Expression in Future Spanish Teachers according to gender and field of study.

In general, women scored higher than men on most items, although statistically significant sex differences were only found in item 7 (*p* < 0.001). Concerning the specialty of studies, the future teachers of early childhood education have more positive perceptions towards CE than the future teachers of primary education since they scored higher on most of the items. Future primary education teachers scored higher than future early childhood education teachers only on item 16, “I prefer Corporal Expression because the students interact more with their classmates than when they do other motor skills content.” It should be noted that statistically significant differences were found according to the specialty of the study and also in numerous items.

Table 3 shows the results obtained in each of the dimensions of the Questionnaire to Assess Perceptions of Corporal Expression in Future Spanish Teachers according to gender and field of study.

Women scored higher than men on Dimension 1 (pleasure) and Dimension 3 (evaluation of CE), while men scored higher on Dimension 2 (preference). However, no statistically significant gender differences were found. For the specialty studied by the participants, the future early childhood education teachers showed a better perception of CE, scoring higher in all three dimensions, and statistically significant differences were found in all of them.

Finally, Cronbach’s alpha was used to calculate the reliability for each of the dimensions of the instrument. Taking Nunally and Bernstein [38] as a reference, satisfactory values were obtained for the three dimensions: a1 = 0.92; a2 = 0.70; a3 = 0.89.

## 4. Discussion

This project was conceived to know the current situation of the perceptions of CE of future teachers during their initial training in the community of Extremadura, Spain. In this sense, a validated questionnaire was used to understand the importance, the value of the rest of the content, and the positive experiences that they have about CE, exploring if the variables gender and educational specialty can be considered as conditioning factors.

In general terms, the future teachers of the Community of Extremadura show good perceptions of CE in most of the items that make up the questionnaire. These results are in agreement with the study developed by González and collaborators [39], in which they found a great interest in CE on the part of university students of early childhood education, even though they had barely received any training in CE. In the same way, a study developed in Romania in which these questions were raised in students of physical education and sports, showed that they had great knowledge and interest in CE even though they had only one subject in the 4 years of university education [40]. Likewise, the future early childhood education teachers showed good perceptions of didactic sequences focused on CE and a great willingness to learn cognitive-social dynamics to guide them [41].

In general, contextual factors such as background or previous experiences, generally higher in girls than in boys, might influence gender disparities. In this sense, other studies have found gender differences in creativity [42], motivation towards CE activities [43], higher personal interest in CE activities [44], or the presence of gender stereotypes [30]. In this research, as mentioned above, women obtained higher scores on most of the items, although significant differences were only obtained in one of them. This is consistent with the results of Vidaci et al. [45], who found higher scores in female university students when analyzing the influences of bodily expression on the development of creative intelligence. However, another Spanish study found no significant differences between genders when it came to assessing the importance of CE content in their initial training [46]. Similarly, no significant differences were found in second-year university students before and after performing motor practices in their initial training [47]. However, regarding the correlation between questionnaire items and trainee instructors’ gender, no apparent differences were discovered.

On the other hand, the educational specialty seems to be a fundamental issue that affects the perceptions that future teachers have towards CE, finding differences both in the items and in the dimensions of the scale, with the future teachers in early childhood education having the highest scores. In this context, there is little literature comparing perceptions of this content according to educational specialty. Current studies already point to the university degree and the university of origin as determining factors in the development of certain content in the classroom by future teachers [48]. For example, Cañadas and coworkers [49] found differences in the perceptions of university students according to the degree they studied, mainly due to the weight of CE throughout their initial training. Likewise, future primary school teachers perceive fear in male students when developing activities related to CE [50], as well as boredom at a general level of all students when proposing dynamics related to CE [51]. On the contrary, university students of early childhood education have very positive attitudes towards CE, demanding a greater weight in the career even though they consider themselves prepared to develop content [39]. Therefore, research suggests developing interventions and programs that improve the perceptions of both prospective teachers and students towards CE at the early childhood [52] and primary school stages [53].

Considering all of the above, there is an evident need to equalize the levels of CE-related content that exist in the different formation specialties, as there are differences in almost all items as well as in the dimensions. This will allow teachers to improve their perceptions of this content, increasing their efforts to implement it in the classroom so that students can benefit from its educational advantages. Primary school teachers, despite having a higher specialization in their initial training, find it much more difficult to implement these activities in the classroom, so new actions must be developed to favour the inhibition of boys and girls during the dynamics.

Therefore, these results may be useful for the review and future modification of the curricula of degree programs that include CE in their initial training, thus trying to broaden and diversify their teaching to reach as many students as possible, since teachers’ perceptions of CE in their training could be fundamental because it will affect their attitudes and self-efficacy, as well as their future employment and their ability to include this content in the educational curriculum. The results of this study are therefore a starting point that will enable teachers and governing bodies to identify the educational needs of the schools in which they work, so that lines of action and collaboration can be established to guarantee the education of their pupils.

Nevertheless, the study has certain limitations, as does any research. The sample was selected through a convenience method, so the results and conclusions drawn should be interpreted with caution. In addition, the entire sample also belongs to the same Autonomous Community, so perceptions may be affected by different socio-demographic variables.

Future research can take many different forms, such as a multicenter investigation to see if regional variation in culture and/or socioeconomic status could affect the outcomes. Similarly, we can observe how the results vary depending on the subject that future teachers will develop or include the educational stage of the Baccalaureate.

## 5. Conclusions

The findings of this study reported significant differences in the perception of CE in prospective teachers according to the specialty of their training, with prospective early childhood education teachers having the best perception of CE compared to primary education teachers. However, no gender differences were found.

In this sense, the educational specialty could be a determinant of prospective teachers’ perceptions of CE in the region of Extremadura, Spain.

Therefore, the equalization of the levels of CE-related content existing in the different educational specialisations could allow teachers to improve their perception of this content, in turn favouring an effort to implement it in the classroom. In short, these results may be useful for the review and future modification of the curricula of degree programs that include CE in their initial training, thus trying to broaden and diversify their teaching to reach as many students as possible, since teachers’ perception of CE in their training may be fundamental because it will affect their attitudes and self-efficacy, as well as their future employment and their ability to include this content in the educational curriculum.

## Figures and Tables

**Table 1:** Sample characterization (N = 437).

Variables	Categories	N	%
Sex	Women	295	67.5
	Men	142	32.5
Educational Specialty	Early Childhood	293	67.0
	Primary School	144	33.0
Variable		Me	SD
Age		22.1	4.4

N: number; %: percentage; Me: median value; SD: standard deviation.

**Table 2:** Scores obtained according to sex and specialty in each of the items of the Questionnaire to Assess Perceptions of Corporal Expression in Future Spanish Teachers. Reproduced with permission from Rojo-Ramos et al., IJERPH; published by MDPI, 2022 [37].

Item	Gender			Specialty		
	Women	Men		Early Childhood	Primary Education	
	M (SD)	M (SD)	*p*	M (SD)	M (SD)	*p*
1. Corporal Expression is useful in teacher training.	4.60 (0.69)	4.50 (0.75)	0.094	4.61 (0.64)	4.38 (0.87)	0.006
2. Corporal Expression allows to express feelings.	4.48 (0.84)	4.56 (0.75)	0.567	4.65 (0.69)	4.29 (0.88)	<0.001
3. The learning received in Corporal Expression is necessary and important.	4.65 (0.62)	4.56 (0.64)	0.081	4.67 (0.58)	4.43 (0.70)	<0.001
4. Corporal Expression classes improve the mood.	4.37 (1.01)	4.22 (0.95)	0.022	4.45 (0.76)	3.91 (1.22)	<0.001
5. Corporal Expression helps to know oneself better, to relate to others and to be creative.	4.62 (0.60)	4.48 (0.65)	0.026	4.66 (0.53)	4.26 (0.76)	<0.001
6. Corporal Expression contributes to global education.	4.47 (0.77)	4.34 (0.83)	0.085	4.53 (0.69)	4.08 (0.95)	<0.001
7. Corporal Expression is good for socialization.	4.73 (0.59)	4.48 (0.69)	<0.001	4.68 (0.51)	4.31 (0.87)	<0.001
8. Corporal Expression is a good social experience and gives you opportunities to get to know your peers in a deeper way.	4.64 (0.63)	4.45 (0.68)	0.002	4.66 (0.55)	4.22 (0.79)	<0.001
9. In the Corporal Expression classes a very positive environment is created.	4.54 (0.77)	4.39 (0.76)	0.012	4.57 (0.69)	4.16 (0.85)	<0.001
10. Corporal Expression provides important relief from accumulated stress.	4.49 (0.76)	4.20 (0.95)	0.003	4.49 (0.72)	3.89 (1.09)	<0.001
11. Corporal Expression also improves overall health and not only physical fitness activities.	4.48 (0.72)	4.29 (0.80)	0.016	4.47 (0.73)	4.10 (0.81)	<0.001
12. The activities taught in Corporal Expression seem important to me.	4.56 (0.72)	4.44 (0.71)	0.035	4.61 (0.60)	4.20 (0.84)	<0.001
13. I like Corporal Expression because it works on aesthetics and social relations.	3.92 (0.98)	3.84 (0.94)	0.321	3.92 (0.96)	3.75 (0.93)	0.033
14. I prefer Corporal Expression to other contents.	3.25 (1.12)	3.33 (1.03)	0.671	3.41 (0.98)	3.08 (1.19)	0.004
15. I prefer Corporal Expression because students interact with their peers more than when doing other motor skills contents.	2.87 (1.27)	2.97 (1.12)	0.309	3.80 (1.05)	2.93 (1.18)	0.849
16. Corporal Expression is more important than the rest of the contents.	3.68 (1.05)	3.70 (1.05)	0.818	2.94 (1.17)	3.49 (1.02)	0.002
17. If doing Corporal Expression in the classes were optional, I would choose to do it.	4.20 (0.88)	4.18 (0.88)	0.778	4.31 (0.78)	3.93 (1.02)	<0.001
18. When I have taken Corporal Expression classes I have liked it because it is something different from what is normally taught.	4.23 (0.93)	4.16 (0.87)	0.250	4.30 (0.81)	3.94 (0.99)	<0.001
19. When I have taken Corporal Expression classes I have liked it because it is cooperative.	4.26 (0.92)	4.21 (0.85)	0.304	4.30 (0.86)	4.06 (0.87)	0.002
20. When I have taken Corporal Expression classes I have enjoyed the time I have spent doing these activities.	4.40 (0.81)	4.20 (0.85)	0.012	4.40 (0.77)	3.98 (0.91)	<0.001
21. When I have taken Corporal Expression classes, I have liked them because they include artistic activities.	3.98 (1.01)	3.85 (1.09)	0.218	3.96 (1.03)	3.74 (1.19)	0.116
22. When I have taken Corporal Expression classes, I have liked them because they involve more games.	4.21 (0.99)	4.17 (0.94)	0.376	4.24 (0.90)	4.06 (1.05)	0.139
23. When I have taken Corporal Expression classes (in my teacher training), I have always wanted more.	4.11 (0.93)	4.02 (0.90)	0.307	4.13 (0.91)	3.88 (0.88)	0.003

M = mean value; SD = Standard deviation. Each score obtained is based on a Likert scale (1–5): 1 “Strongly disagree”, 2 “Disagree”, 3 “Indifferent”, 4 “Agree”, 5 “Strongly agree”.

**Table 3:** Descriptive analysis and differences for each dimension of the questionnaire.

	Total	Gender			Specialty		
Dimensions	M (SD)	Men	Women	*p*	Early Childhood	Primary Education	*p*
Evaluation of CE	3.31 (0.56)	4.36 (0.5)	4.50 (0.4)	0.016	4.53 (0.64)	4.15 (0.67)	<0.001 *
Preference	3.31 (0.87)	3.33 (0.83)	3.26 (0.94)	0.671	3.38 (0.84)	3.16 (0.92)	0.002 *
Pleasure	4.13 (0.72)	4.11 (0.69)	4.19 (0.77)	0.069	4.23 (0.65)	3.94 (0.80)	<0.001 *

*: *p* is significant at leven *p* < 0.016. CE: Corporal Expression; M = mean value; SD = Standard Deviation. Each score obtained is based on a Likert scale (1–5): 1 “Strongly disagree”, 2 “Disagree”, 3 “Indifferent”, 4 “Agree”, 5 “Strongly agree”.

## Data Availability

The datasets used during the current study are available from the corresponding author on reasonable request.

## Appendix A

**Table A1:** Questionnaire to Assess Perceptions of Corporal Expression (Cuestionario para la Evaluación de la percepción hacia la Expresión Corporal). Reproduced with permission from Rojo-Ramos et al., IJERPH; published by MDPI, 2022 [37].

1. La Expresión Corporal es útil en la formación del profesorado.
2. La Expresión Corporal permite expresar los sentimientos
3. Los aprendizajes que se reciben en Expresión Corporal son necesarios e importantes.
4. Las clases de Expresión Corporal mejoran el estado de ánimo
5. La Expresión Corporal ayuda a conocerse mejor, a relacionarse con los demás y a ser creativo.
6. La Expresión Corporal contribuye a la educación global.
7. La expresión corporal es buena para socializarse.
8. La Expresión Corporal es una buena experiencia social y te da la oportunidad de conocer a tus compañeros de una manera más profunda
9. En las clases de Expresión Corporal se crea un ambiente muy positive
10. La Expresión Corporal proporciona un importante alivio del estrés acumulado.
11. La Expresión Corporal también mejora la salud a nivel general y no solo las actividades de condición física
12. Las actividades que se enseñan en Expresión Corporal me parecen importantes.
13. Me gusta la Expresión Corporal porque trabaja la estética y las relaciones sociales
14. Prefiero la Expresión Corporal a otros contenidos.
15. La expresión Corporal es más importante que el resto de los contenidos.
16. Prefiero la Expresión Corporal porque los alumnos interactúan más con sus compañeros que cuando hacen otros contenidos de motricidad.
17. Si hacer Expresión Corporal en las clases fuera opcional, elegiría hacerla.
18. Cuando he realizado clases de Expresión Corporal, me han gustado porque es algo diferente a lo que se normalmente se enseña.
19. Cuando he realizado clases de Expresión Corporal, me han gustado porque se trabaja la cooperación.
20. Cuando he realizado clases de Expresión Corporal, me ha gustado el tiempo que he dedicado a estas actividades.
21. Cuando he tomado clases de Expresión Corporal, me han gustado porque incluyen actividades artísticas.
22. Cuando he tomado clases de Expresión Corporal, me han gustado porque incluyen más juegos.
23. Cuando he tomado clases de Expresión Corporal (en mi formación docente), siempre he querido más.
